# Metabolic responses of eukaryotic microalgae to environmental stress limit the ability of FT-IR spectroscopy for species identification

**DOI:** 10.1016/j.algal.2015.06.009

**Published:** 2015-09

**Authors:** Thomas Driver, Amit K. Bajhaiya, J. William Allwood, Royston Goodacre, Jon K. Pittman, Andrew P. Dean

**Affiliations:** a Faculty of Life Sciences, The University of Manchester, Michael Smith Building, Oxford Road, Manchester M13 9PT, UK; b School of Chemistry, Manchester Institute of Biotechnology, The University of Manchester, 131 Princess Street, Manchester M1 7DN, UK; c Environmental & Biochemical Sciences Group, The James Hutton Institute, Invergowrie, Dundee, DD2 5DA Scotland, UK; d Department of Geography, University of Sheffield, Sheffield S10 2TN, UK

**Keywords:** Microalgae, FT-IR spectroscopy, Species discrimination, Nutrient limitation

## Abstract

Fourier Transform Infrared (FT-IR) spectroscopy is a robust method for macromolecular analysis and differentiation of microorganisms. However, most studies are performed in controlled conditions and it is unclear whether this tool is appropriate for the identification of eukaryotic microalgae species from variable environments. In order to address this, nine closely-related species of marine and freshwater microalgae were grown under controlled (non-stressed) and variable (non-stressed and stressed) conditions, including nutrient-stressed and wastewater-stressed conditions. Following optimization of data processing methods, FT-IR spectra from all species and conditions were compared. The substantial metabolic changes that were caused by nutrient starvation restricted the ability of FT-IR spectroscopy to differentiate the microalgal species grown under variable conditions efficiently. Comparison of unsupervised and supervised multivariate data analysis methods found that principal component-discriminant function analysis was able best to differentiate between some species under controlled conditions but still gave poor differentiation under variable environmental conditions.

## Highlights

• FT-IR spectroscopy can be used to generate metabolic fingerprints in algae.
• Metabolic fingerprinting has been proposed to differentiate species and strains.
• Algae exhibit high metabolic plasticity in response to environmental change.
• This restricts the use of FT-IR spectroscopy as a discriminatory tool.
• Discrimination is improved by multivariate statistical analysis techniques.

## 1 Introduction

**Fig. 1:**
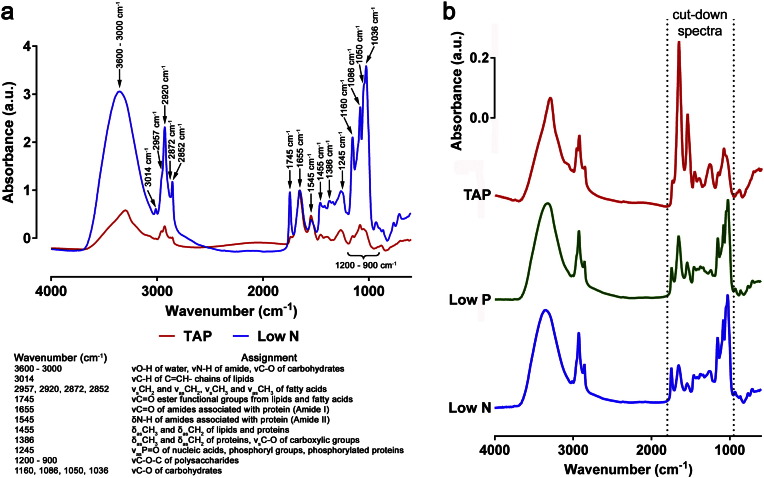
FT-IR spectra from *C. reinhardtii*. Typical FT-IR spectra (wavenumbers 4000–600 cm^− 1^) *C. reinhardtii* grown in either standard TAP or Low N TAP media (a). Both spectra are normalized to the amide I peak at 1655 cm^− 1^. Major band assignments, including bands associated with lipids, proteins and carbohydrates, are indicated by arrows and defined in the list below the graph. Comparison of averaged spectra, following EMSC2 normalisation, generated from *C. reinhardtii* grown in TAP (from 18 replicates), Low P TAP (from 18 replicates), or Low N TAP media (from 9 replicates) (b). The spectral region referred to as cut-down spectra (wavenumbers 1800–950 cm^− 1^) in subsequent experiments is indicated by dotted lines.

Fourier Transform Infrared (FT-IR) spectroscopy is a powerful and potentially under-utilised tool for many aspects of microalgal research, including metabolic fingerprinting of microalgal strains. These organisms are of increasing interest as sustainable sources of various high-value chemicals and products. For example, many algal strains can produce large amounts of the neutral lipid triacylglycerol, which could be converted to biodiesel through industrial transesterification [1]. At present, this is not economically viable and research is needed to screen for and develop high lipid producing strains, and improve the methods of algal growth [2]. FT-IR spectroscopy is an extremely useful technique for such research either to compare rapidly metabolic fingerprints in different algal species/strains or in strains from different growth conditions [3], [4], [5], [6]. Whole algal cells can be analysed by FT-IR spectroscopy, enabling the production of detailed spectra without the need to perform any complex and time consuming cell extractions. Chemical bonds within functional groups of biochemical molecules have distinct vibrational properties, allowing the relative amounts of these biochemical macromolecules to be effectively determined through FT-IR spectral analysis [7]. Thus, the analysis of these spectra can provide important biochemical information, such as the relative quantification of carbohydrates, lipids and proteins found within a sample (Fig. 1a).

While FT-IR spectroscopy could certainly be a useful tool for bioprospecting novel algal strains for biotechnological applications by high-throughput metabolic fingerprinting [6], it has been suggested that the technique could also be used to discriminate and identify different species of algae present in a sample taken directly from the environment [8]. A large body of research has clearly demonstrated that FT-IR spectroscopy can be successful in the identification of many microorganisms, particularly bacterial and fungal species [9], [10], [11], [12], [13]. The identification of five cyanobacteria (blue-green algae) species has been achieved with some success using supervised statistical methods [14]. However, that study was performed using controlled laboratory conditions for the cultivation of the strains and a single type of nutrient-rich media, therefore its success cannot be extrapolated to species classification under less controlled conditions. In addition, species classification is likely to be considerably more difficult when the number of species tested is higher. More recent studies examining cyanobacteria and eukaryotic algae have used a higher number of species grown to different growth stages or in two types of media containing different N sources (nitrate or ammonium) [15], [16], [17]. Results again showed a high percentage of correct species classification, although it could still be argued that these studies cultivated strains under very controlled and uniform conditions. However, it is less clear if the identification of eukaryotic microalgae species grown under variable conditions would be as efficient using FT-IR spectroscopy.

Eukaryotic microalgae are arguably more complex microorganisms than bacteria and yeast. They contain a large metabolically active chloroplast that accounts for a significant proportion of each cell's volume, which has been considered to make species identification through FT-IR spectroscopy more challenging [16]. In addition to this, microalgae have considerable phenotypic plasticity in how they respond to environmental stimuli such as N starvation and salt stress [18], [19]. As a result, individual species of microalgae produce very different FT-IR spectra under different environmental conditions [6], [20]. Thus the development of a spectral library for microalgal species identification may be troublesome, as each species is unlikely to have a single characteristic FT-IR spectrum to use as a guide.

This study therefore aimed to evaluate whether nutrient availability affect the ability of FT-IR spectroscopy to classify eukaryotic microalgal species correctly. To achieve this, optimum data processing methods were determined using data from *Chlamydomonas reinhardtii* and a meta-analysis of *C. reinhardtii* studies performed under different conditions. These data processing methods were then applied to nine different chlorophyte species grown in a number of different media.

## 2 Materials and methods

### 2.1 Microalgal strains and growth conditions

Microalgal strains were originally obtained from the UK Culture Collection of Algae and Protozoa (CCAP), Oban, Scotland, UK or the Plymouth Algal Culture Collection (PLY), Plymouth, UK: *Chlamydomonas concordia* (PLY 491), *Chlamydomonas debaryana* (CCAP 11/70), *C. reinhardtii* (CCAP 1132C), *Chlorella luteoviridis* (CCAP 211/3), *Chlorella vulgaris* (CCAP 211/79), *Desmodesmus intermedius* (CCAP 258/38), *Dunaliella tertiolecta* (PLY 83), *Hindakia tetrachotoma* (CCAP 222/81) and *Parachlorella kessleri* (CCAP 211/11G). All are freshwater species apart from *C. concordia* and *D. teriolecta*, which are marine species.

*C. reinhardtii* was grown photo-heterotrophically in batch cultures of Tris–acetate–phosphate (TAP) medium, which includes 7 mM N (as NH_4_Cl) and 1 mM P (as K_2_HPO_4_/KH_2_PO_4_) [21], and in modified TAP media with reduced concentrations of N (0.7 mM in Low N TAP) or P (0.01 mM in Low P TAP). In Low P TAP the K concentration was maintained by adding KCl. *C. concordia* was grown in TAP medium but with an addition of 30 g/L NaCl (~ 0.5 M). *D. tertiolecta* was grown in ASP2 medium, an enriched artificial seawater medium, which includes 18 g/L NaCl, 0.59 mM N (as NaNO_3_), 29 μM P (as K_2_HPO_4_) and 9 μM Fe (as FeCl_3_) [22]. Cultures were also grown in modified ASP2 media with reduced concentrations of N (0.12 mM in Low N ASP2) or P (2.9 μM P in Low P ASP2), or a Fertilised-ASP2 medium with 5-fold increased concentrations of P (145 μM), N (2.95 mM) and Fe (45 μM). Cultures were grown for 20 days until stationary phase in triplicate under environmentally controlled conditions on an orbital shaker (120 rpm) at 25 °C with a 16 h light:8 h dark light regime, and a photon flux of either 100 μmol m^− 2^·s^− 1^ (for *D. tertiolecta* cultures) or 150 μmol m^− 2^·s^− 1^ (for *C. reinhardtii* and *C. concordia* cultures). Acclimated and non-acclimated strains of *C. debaryana*, *C. luteoviridis*, *C. vulgaris*, *D. intermedius*, *H. tetrachotoma* and *P. kessleri* were grown for 10 days in municipal secondary-treated wastewater in a previous experiment [3]. Overall, there were six independent *C. reinhardtii* growth experiments, each with multiple replicates and run under the same conditions at six different times, with collection of FT-IR spectra carried out separately after each experiment. In addition, there were single growth and FT-IR spectroscopy experiments for *C. concordia* and *D. tertiolecta*, also each carried out at different times. In addition to the majority of the data specifically acquired for this study, the spectral data of the wastewater-grown algae had been previously collected and published [3].

### 2.2 FT-IR spectroscopy

All FT-IR spectra collection was performed using the same methodology and using the same FT-IR instrument. A 1 mL sample was taken from each flask for centrifugation at 14000 *g* for 5 min. The supernatant was discarded and pelleted wet biomass was weighed. Each sample was then normalised to a concentration of 60 mg mL^− 1^ with Milli-Q (Millipore) water. 30 μL of each sample was pipetted onto a well of a 96-well silicon microplate and dried at 40 °C overnight. Spectra were collected using an FT-IR spectrometer (Bruker Equinox 55 FT-IR spectrometer), equipped with a deuterated triglycerine sulphate detector, and using an HTS-XT high-throughput microplate extension. A spectral range of 4000–600 cm^− 1^ was collected, with four scans co-added for each spectra.

### 2.3 Comparison of spectral ranges

Resulting spectra were imported into MATLAB v.2010a (MathWorks) and normalised using the extended multiplicative scatter correction type two method (EMSC2) [23]. Normalised spectra were then imported into The Unscrambler v.10.1 (CAMO Software). Principal Component Analysis (PCA) was then performed over two separate spectral ranges: full spectra (4000–600 cm^− 1^) and cut-down spectra (1800–950 cm^− 1^) which contained the majority of the spectral information. Resulting PCA scores and loading plots were compared to determine the optimum spectral range for microalgal metabolic fingerprinting. PCA was applied as a non-supervised model that is appropriate since it is representative of near to the full variance within the dataset. Clustering indicates which experimental factors are the sources of greatest variance within the data. For all analyses plots were generated using Prism v6 (GraphPad Software).

### 2.4 Comparison of data processing methods

The results of three data processing options were compared. EMSC2 normalisation on the raw FT-IR data as described above was compared to 1st derivative and 2nd derivative spectra. Derivatised spectra were generated from raw spectral data using The Unscrambler v.10.1 using a Savitzky–Golay algorithm before EMSC2 normalisation in MATLAB. PCA was then performed on the three separate data sets and comparisons between the data processing methods were made.

### 2.5 Additional data analysis

Lipid content and carbohydrate content was estimated through measuring individual band heights for total lipid (1740 cm^− 1^), amide I (1655 cm^− 1^) and carbohydrate (1160, 1086, 1050 and 1036 cm^− 1^) (see Fig. 1a) and calculating lipid:amide I peak height ratios and carbohydrate:amide I peak height ratios; calculations were performed in MATLAB. Partial Least Squares Regression (PLSR) analysis was performed in The Unscrambler. PLSR values were calculated using the more conservative method using cross validation. PLSR is a classic method of multivariate prediction analysis and so was applied in this case. Principal Component-Discriminant Function Analysis (PC-DFA) was performed in MATLAB. PC-DFA is appropriate as a supervised model in this case since it is based on an *a priori* PCA performed in step 1. PLS-DA or OPLS-DA are alternatives, however since they are based on an *a priori* PLS rather than PCA, they cannot be related back to the PCs from the PCA performed in step 1. Supervised methods such as OPLS-DA are perhaps more likely to over fit the data than PC-DFA.

## 3 Results and discussion

### 3.1 Comparison of FT-IR spectral range and data-processing methods

**Fig. 2:**
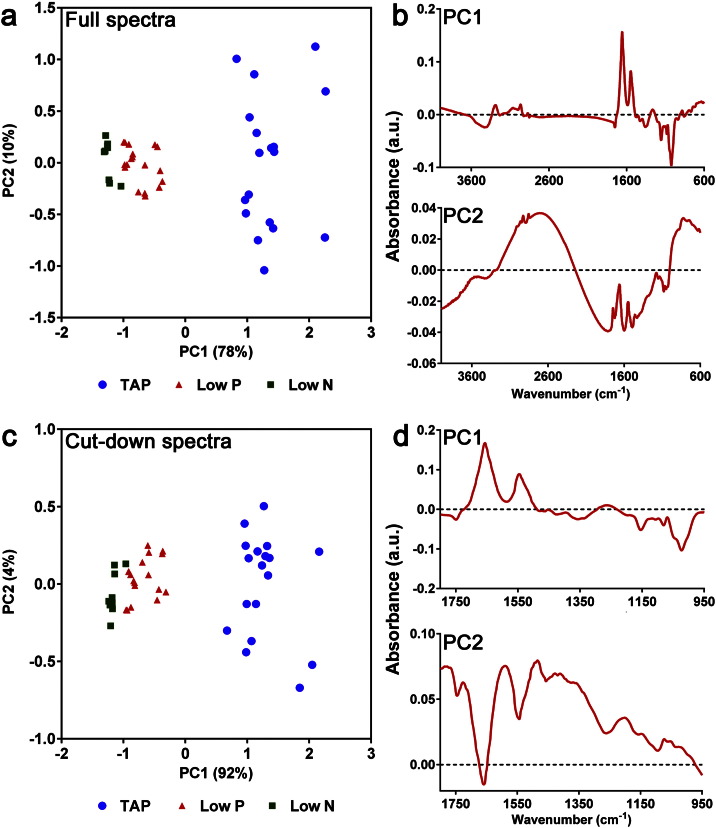
Comparison of the effect of full or cut-down FT-IR spectral ranges on sample clustering. PCA scores plots (a) and PC1 and PC2 loading plots (b) for full spectra (wavenumbers 4000–600 cm^− 1^) and scores (c) and loading plots (d) for cut-down spectra (wavenumbers 1800–950 cm^− 1^) generated from *C. reinhardtii* grown in either standard TAP (18 replicates), Low P TAP (18 replicates), or Low N TAP media (9 replicates).

We first ascertained whether use of the full FT-IR spectral range or specific regions of the spectrum could affect the clustering and discrimination of spectra derived from microalgae grown under non-stressed and various nutrient stressed conditions. Furthermore, the spectra for this analysis were derived from *C. reinhardtii* samples obtained from six identical but independent experiments performed at different times. A number of specific bands in the FT-IR spectra can be associated with specific macromolecules and metabolites, many of which are clearly visible in response to stress, such as N limitation (Fig. 1a). While there are many discernible bands of interest across the full spectra (wavenumbers 4000–600 cm^− 1^), many key bands can be found within a fingerprint region of so called ‘cut-down’ spectra (wavenumbers 1800–950 cm^− 1^) (Fig. 1b). PCA plots and loading plots were generated for both full spectra (Fig. 2a and b) or cut-down spectra (Fig. 2c and d) of *C. reinhardtii* grown for 7 days until late exponential phase in nutrient replete (non-stressed) TAP medium and in N or P limited (Low N and Low P TAP) media (Fig. 1b). For both the full spectra and cut-down spectra there was clear clustering between stressed and non-stressed treatments along PC1 in each PCA scores plot and also some discrimination between Low-N and Low-P treatments (Fig. 2a and c). Because this non-supervised PCA method separated the treatments very clearly, a potentially more successful supervised PC-DFA method was not needed. Indeed, a PC-DFA scores plot of the same spectra was essentially identical to the PCA plot (data not shown). According to the PC1 loadings plots, the differences between the stressed and non-stressed treatments were determined by peak changes mainly within the 1800–950 cm^− 1^ region (the cut-down spectra) with little spectral change within the 4000–1800 cm^− 1^ region (Fig. 1b). Thus, the Low-N and Low-P treatments were characterized by an increase in lipid and carbohydrate as well as a relative decrease in protein for both sets of spectra (Fig. 2b and d). However, the PC2 loadings varied considerably between the two sets of spectra, with PC2 of the full spectra being characterized by a significant amount of non-biological data, principally *v*O–H stretching of water within the wavenumber range 3639–3029 cm^− 1^ (Fig. 2b). In addition, in the full spectra, PC2 accounts for 10% of total variation (Fig. 2b), compared to 4% in the cut-down spectra (Fig. 2d). As a result, there is more variation accounted for by non-biological data when the full range of the FT-IR spectra is used.

There are arguments for and against the use of full or ‘cut-down’ spectra for microalgal FT-IR spectroscopy analysis. Previous studies have used full spectral ranges [15], whilst others have used cut-down spectra [17], [24]. Results from this study clearly show that the cut-down spectra enabled more efficient clustering of different groups by PCA (Fig. 2), and that the clustering was based on more biologically relevant information. One argument against the use of a reduced spectral range is that some metabolic information is lost, such lipid associated *v_s_*CH_2_ and *v*_as_CH_2_ bands of associated with lipids (*ca.* 3000–2800 cm^− 1^) and the region associated with *v*N–H and *v*O–H stretching (*ca.* 3600–3000 cm^− 1^) (Fig. 1a). Nevertheless, the analysis here suggests that whilst there is more information available in the full spectra, this information was not as important to discriminate between different treatment groups of microalgae in this study. As a result the cut-down spectra of 1800–950 cm^− 1^ was used for the remainder of analysis in this study. A previous study evaluating six cyanobacteria species found that use of the same cut-down spectral region in comparison to the full spectrum or more discrete spectral windows was the most appropriate for discriminating between all six species [17].

Many previous FT-IR spectroscopy studies have used different data pre-processing methods before spectral analysis by clustering. We also investigated the effect of different processing methods on PCA clustering of the spectra derived from *C. reinhardtii* grown in standard TAP, and Low P or Low N TAP media. Cut-down spectra (wavenumbers 1800–950 cm^− 1^) were processed using EMSC2 normalisation without derivatisation (Fig. S1a, b), spectra converted to their 1st derivative before EMSC2 normalisation (Fig. S1c, d), and spectra converted to their 2nd derivative before EMSC2 normalisation (Fig. S1e, f). There was very little difference between the resulting scores plots with each plot showing clear clustering between stressed and non-stressed samples, and equivalent differentiation between Low-P and Low-N samples. As a result EMSC2 normalisation without derivatisation was used for species analysis as it is the closest to raw spectral data and there is less risk of important biological data being removed through data processing.

The use of spectra derived from six independent but identical experiments also allowed an assessment of the variability between separate experiments and whether individual experiments performed at different times could be distinguished by FT-IR spectroscopy. By plotting the data shown in Fig. 2c on the basis of experiment, it could be shown that clustering of non-stressed (standard TAP) and nutrient stressed (Low-P and Low-N TAP) samples was good even though spectral data was derived from different experiments (Fig. S2). Whilst there was some variation accounted for by experimental timing, such as some variation from Experiment 6 (Fig. S2b, c), these differences do not affect the ability of PCA to distinguish between samples grown in different nutrient replete and nutrient limited media. It should be noted that all six experiments were performed under environmentally controlled conditions with parameters such as light intensity and temperature as identical as possible. This suggests that if a standard metabolic fingerprint is obtained, such as a species-specific fingerprint, with a high level of experimental control species classification may be possible, depending on whether species-dependent characteristics can be determined from FT-IR spectral information.

### 3.2 FT-IR spectroscopy can discriminate responses to nutrient limitation in a marine microalga

**Fig. 3:**
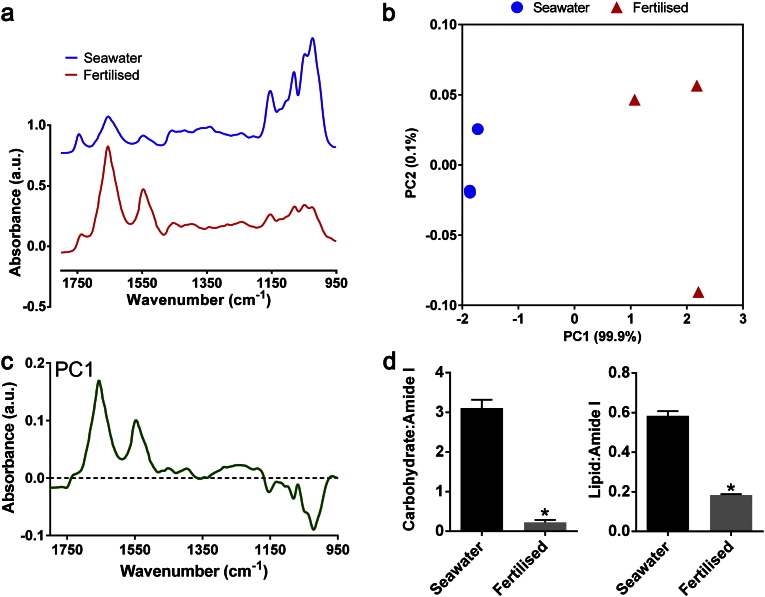
An example of nutrient stress-induced metabolic response determined by FT-IR spectroscopy in a marine microalga. Mean EMSC2 normalised FT-IR spectra (wavenumbers 1800–950 cm^− 1^) generated from *D. tertiolecta* grown in artificial seawater (ASP2) media or fertilised seawater media (a). PCA score plot (b) and PC1 loading plot (c) derived from the replicate spectra. Carbohydrate:amide I and lipid:amide I peak height ratio values generated from replicate spectra (d). Data are mean values ± SE of three replicates. Significant differences (p < 0.05) are indicated with an asterisk.

To demonstrate the significant metabolic plasticity that eukaryotic microalgae, including a marine microalga, can display in response to an environmental stress such as nutrient starvation, analysis was performed using the marine alga *D. tertiolecta*. *D. teriolecta* cultures grown for 20 days until late exponential phase in artificial seawater (ASP2) medium were compared to those cultivated in fertilised seawater medium. As would be expected, growth of the cultures was significantly increased following fertilisation with an approximately 2.5-fold increase in wet weight biomass (data not shown). The EMSC2-normalised FT-IR spectra showed that the metabolic fingerprint of *D. tertiolecta* varies markedly when grown in the different media (Fig. 3a), while the PCA scores plot generated from these spectra showed distinct clustering of spectra derived from ASP2-grown cells from those grown in fertilised medium along PC1 (Fig. 3b). The PC1 loading plot suggests that ASP2-grown nutrient-stressed cells are clustered towards the negative side of the PC1 axis as a result of having higher (negative on loading plot) carbohydrate peaks (*v*C–O of carbohydrates at 1160, 1086, 1050 and 1036 cm^− 1^) and a total lipid peak (*v*C=O of ester functional groups from lipids and fatty acids at 1745 cm^− 1^), as well as a reduction (positive on loading plot) in protein peaks (*v*C=O of amides associated with proteins; amide I at 1655 cm^− 1^ and δ N–H of amides associated with protein; amide II at 1545 cm^− 1^) (Fig. 3c). Relative quantification of carbohydrate and lipid content by calculating carbohydrate:amide I and lipid:amide I peak height ratio values (Fig. 3d) demonstrated that both metabolic classes are significantly higher in the nutrient stressed seawater grown cells than in fertiliser-grown cells.

Previously we have demonstrated that metabolic responses to N or P limitation, notably carbohydrate and lipid induction, can be clearly determined by FT-IR spectroscopy for two freshwater microalgae species, *C. reinhardtii* and *Scenedesmus subspicatus* [5], [6], [25]. Here we show that a marine microalga displays a similar nutrient limitation response, which elicits a substantial change in FT-IR spectra, and thus two very distinct spectra can be generated from the same species cultivated in just two different conditions. This is similar to N limitation responses detected by FT-IR spectroscopy analysis described previously for the related marine alga *Dunaliella salina* [26].

### 3.3 FT-IR analysis of nine microalgal species grown in stressed and non-stressed conditions

**Fig. 4:**
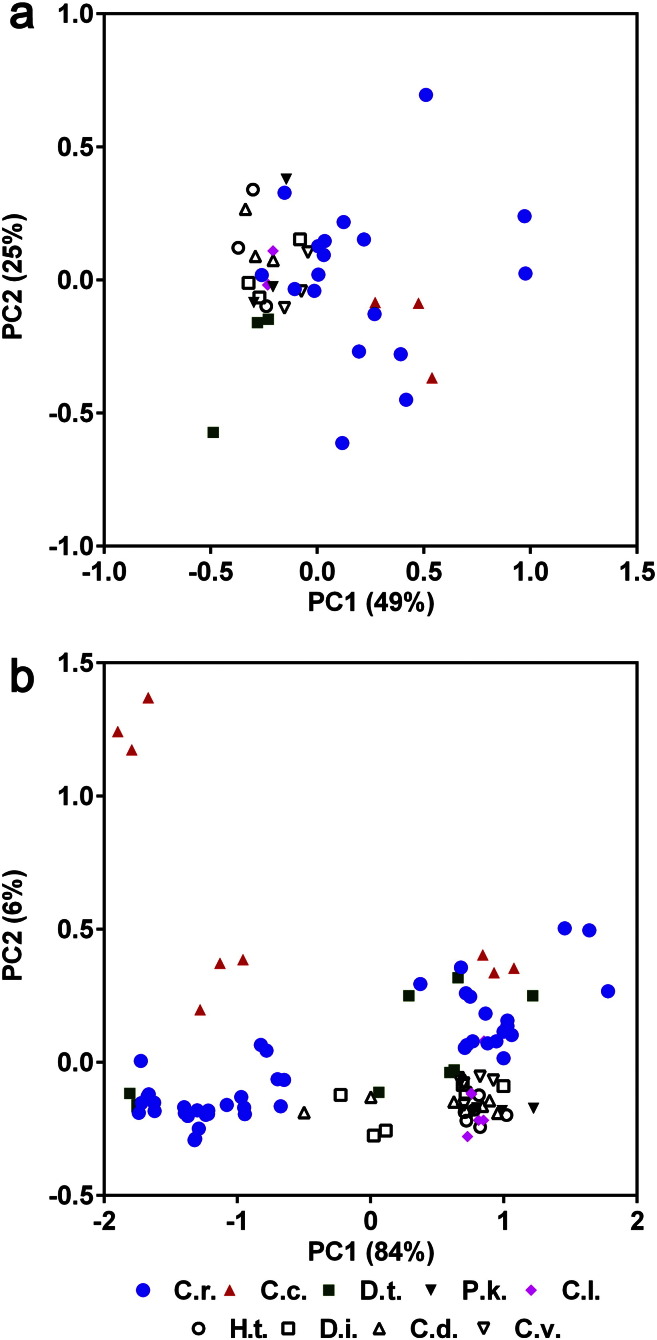
PCA score plots of EMSC2 normalised cut-down FT-IR spectra of nine algal species grown for 7 days in non-stressed conditions (a) and a combined plot of the species grown in non-stressed and stressed conditions (b). Different species are highlighted using different symbols. C.r. = *C. reinhardtii*, C.c. = *C. concordia*, D.t. = *D. tertiolecta*, P.k. = *P. kessleri*, C.l. = *C. luteoviridis*, H.t. = *H. tetrachotoma*, D.i. = *D. intermedius*, C.d. = *C. debaryana*, C.v. = *C. vulgaris.* Mean spectra for each species is shown in Fig. S3.

Using the data processing methods chosen for the *C. reinhardtii* data, this alga was directly compared with eight other eukaryotic microalgae species, including *D. tertiolecta* and another marine alga *C. concordia*, plus six other freshwater species, under conditions classified as stressed or non-stressed, in order to assess the prospects of eukaryotic microalgae species classification by FT-IR spectroscopy. For *C. reinhardtii*, *C. concordia* and *D. tertiolecta* the stressed and non-stressed conditions were nutrient starved and nutrient replete conditions, respectively. For *C. debaryana*, *C. luteoviridis*, *C. vulgaris*, *D. intermedius*, *H. tetrachotoma* and *P. kessleri*, the stressed and non-stressed conditions were dependent on whether the strains were non-acclimated (by never previously being exposed to raw municipal wastewater) or acclimated (after 8 weeks exposure), respectively to growth in municipal wastewater, as described previously [3]. The mean spectra for each species and growth condition are shown in Fig. S3. PCA scores plots of EMSC2 normalised cut-down FT-IR spectra were generated for the nine algal species grown in non-stressed conditions (Fig. 4a) and a combined plot of the species grown in non-stressed and stressed conditions (Fig. 4b). As was expected from the analysis of *C. reinhardtii* (Fig. 2) and *D. tertiolecta* (Fig. 3) separately, the degree of individual species clustering was greater in non-stressed conditions than in the combined plot of the species grown in all conditions (Fig. 4). Nevertheless, even for the non-stressed samples, individual species could not be easily discerned by PCA. In particular, there was considerable overlap of the *C. reinhardtii* samples with the clusters of other adjacent species (Fig. 4a). *C. reinhardtii* is the only species that has been analysed over numerous separate experiments and therefore the larger sample size would likely have exacerbated the variation within the *C. reinhardtii* samples, which is almost as great as the variation between all other species. Thus, due to the similarity of the metabolic fingerprints from these microalgae species, the experimental-derived variation for the *C. reinhardtii* samples may affect the ability to differentiate between all the species, even when grown under non-stressed conditions.

Previous studies have shown that under very controlled conditions, the identification of eukaryotic microalgae by using FT-IR spectroscopy can be possible [15], [16]. However, it was not clear whether the same level of classification would be gained with highly similar species grown under a number of different conditions. When the added complexity of stress-inducing conditions was added, species clustering was even poorer when analysed by PCA. For example, there are three separate clusters for *C. concordia* within the PCA scores plot (Fig. 4b), which can be accounted for by the three different conditions (TAP, Low-P TAP and Low-N TAP) that this alga was grown in (Fig. S4). Similar variation was seen for most of the other species, and once again, the variation within the *C. reinhardtii* samples was considerable (Fig. 4b).

The ability of FT-IR spectroscopy to discriminate individual species under either controlled (non-stressed) or variable (combined non-stressed and stressed) conditions was further quantified by PLSR analysis. This analysis assessed the ability of a PLS model to separate each species. The PLS scores plots used to generate the models are shown in Fig. S5, and like the PCA plots, show the poor separation of each species. A regression slope gradient value was calculated for each species under both the non-stressed or combined stressed and non-stressed conditions (Table 1). Only the *C. reinhardtii* non-stressed model gave a regression value closest to 1 and thus indicates that the model would be moderately successful at discriminating this species from the rest from this spectral data set. However, the model for *C. reinhardtii* is considerably weaker using the combined stressed and non-stressed data set as the FT-IR spectra are significantly more variable. The values for all other species models are very low (< 0.2), confirming the inability to discriminate individual species. The distinction between *C. reinhardtii* and the other species PLSR scores may be partly due to sample number for each species used in this analysis. Here 45 individual *C. reinhardtii* samples were used in the full dataset, whereas for some other species like *C. vulgaris* or *C. luteoviridis* there were only six samples. An analysis of *Actinobacteria* found that up to 15 replicate strains per species were needed in a reference dataset to obtain 80–90% identification rates, while for some more closely related bacterial species, higher numbers of strains were needed [27], [28].

**Table 1:** PLS regression values of EMSC2 normalised, cut-down FT-IR spectra for nine algal species grown in stressed and/or non-stressed conditions.

Species	PLS regression slope gradient	
	Non-stressed media	Non-stressed and stressed media
*C. concordia*	0.1404	0.1742
*C. debaryana*	0.0551	− 0.0005
*C. luteoviridis*	− 0.0029	0.1955
*C. reinhardtii*	0.6814	0.3845
*C. vulgaris*	0.0109	0.1346
*D. intermedius*	0.0112	0.0224
*D. tertiolecta*	0.1432	0.0327
*H. tetrachotoma*	0.0492	0.0308
*P. kessleri*	0.0702	0.0778

These data show that the ability to classify species identity using FT-IR spectra is further complicated when growth conditions are not strictly controlled and demonstrates the challenges of phenotypic variance for microalgae. With substantial physiological and metabolic changes occurring within the microalgal cell when nutrient availability differs, including the large induction of carbohydrates (principally as starch) and lipids (principally as triacylglycerol) (Fig. 1), resulting FT-IR spectra will also change markedly, and as a result the classification of an individual species from a mixed sample is considerably more challenging. Therefore FT-IR spectra alone may be insufficient to separate such closely-related species unless the environmental conditions used for growth are carefully controlled.

### 3.4 Evaluation of supervised statistical analysis for species classification

**Fig. 5:**
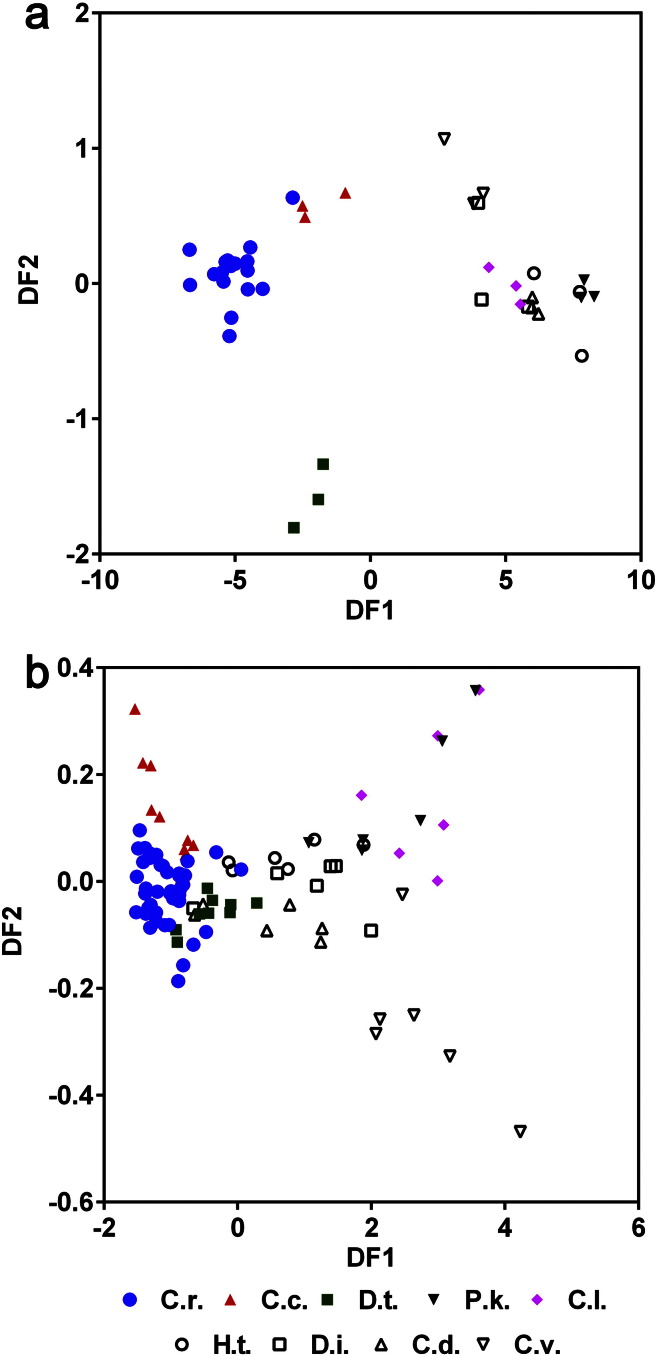
PC-DFA scores plots of EMSC2 normalised cut-down FT-IR spectra of nine algal species grown for 7 days in non-stressed conditions (a) and a combined plot of the species grown in non-stressed and stressed conditions (b). Different species are highlighted using different symbols as described in Fig. 4.

Supervised data analysis methods have been commonly used for species identification [29]. As PCA was unable to distinguish between the nine species, the supervised PC-DFA statistical method was examined to see if better clustering of these species could be achieved. Comparison of PC-DFA scores plots with the PCA scores plots shown in Fig. 4 demonstrates that clustering of individual species was much stronger using PC-DFA (Fig. 5). *C. reinhardtii*, *C. concordia* and *D. tertiolecta* samples from the non-stressed growth conditions could be more easily distinguished from each other and from the other six species samples, which still overlap (Fig. 5a). However, although individual species clustering from the combined non-stressed and stressed data set was improved considerably by PC-DFA compared to PCA, there was still considerable overlap between some samples (Fig. 5b). This indicates that there may be some regions within the spectra that are characteristic for some of the species but not all. It is particularly noticeable that despite the marked differences within *C. reinhardtii* and *D. tertiolecta* spectra under nutrient replete and nutrient starved conditions (Fig. 1, Fig. 3) all samples from each of these species can be tightly grouped by PC-DFA. The main conclusion from this study is that large stress-induced variation in metabolic fingerprints in closely related eukaryotic microalgae restricts the ability of analytical techniques such as FT-IR spectroscopy to differentiate individual species. However, the PC-DFA results indicate that further development of supervised methods of data processing and data analysis, in combination with the generation of large species reference datasets, may allow more efficient discrimination of closely related microalgae from non-controlled environmental samples in the future.

## 4 Conclusion

FT-IR spectroscopy is a highly informative and robust tool for the analysis of microalgae in controlled environments. High phenotypic plasticity of eukaryotic microalgae in response to environmental conditions such as nutrient limitation results in substantial macromolecular and metabolic change that is easily recorded within an FT-IR spectrum. Previous studies have demonstrated the ability of FT-IR spectroscopy to differentiate and thus identify individual species of cyanobacteria in controlled conditions. This study suggests that the substantial metabolic changes that result from environmental variability restricts the ability of FT-IR spectroscopy to differentiate closely related eukaryotic microalgae efficiently when they are cultured under variable conditions or taken from natural systems.

## Author contributions

R.G., J.K.P. and A.P.D. designed the research, T.D., A.K.B., J.W.A. and A.P.D. performed the research, T.D., A.K.B., J.W.A., R.G., J.K.P. and A.P.D. analysed the data and wrote the paper.
