# Supplementary material for: Metabolic responses of eukaryotic microalgae to environmental stress limit the ability of FT-IR spectroscopy for species identification
Source: Algal Res. 2015 Sep;11:148–55. doi: 10.1016/j.algal.2015.06.009 (PMC4694091; doi:10.1016/j.algal.2015.06.009)
Supplement: Supplementary file 1 — Supplementary figures. [file mmc1.pdf]

Supplementary Figure 1

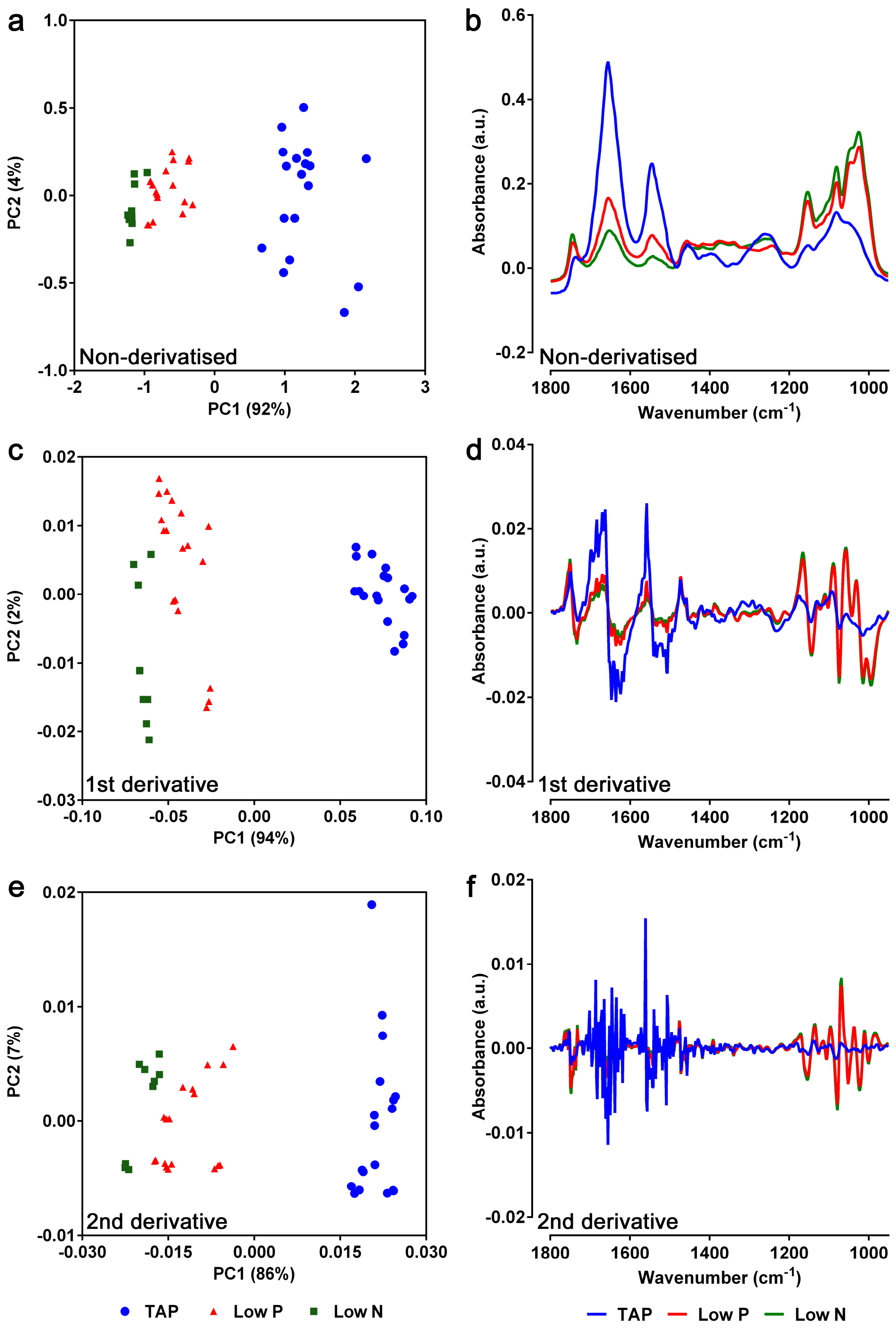

Fig. S1. Comparison of the effect of different data processing methods of FT-IR spectra on sample clustering, using *C. reinhardtii* grown in either standard TAP, Low P TAP, or Low N TAP media. PCA score plots of cut-down spectra processed using EMSC2 normalisation without derivatisation (a, b), spectra converted to their 1st derivative before EMSC2 normalisation (c, d), and spectra converted to their 2nd derivative before subsequent EMSC2 normalisation (e, f). Mean spectra from 9 or 18 replicates for each growth treatment is shown in panels (b), (d) and (f).

Supplementary Figure 2

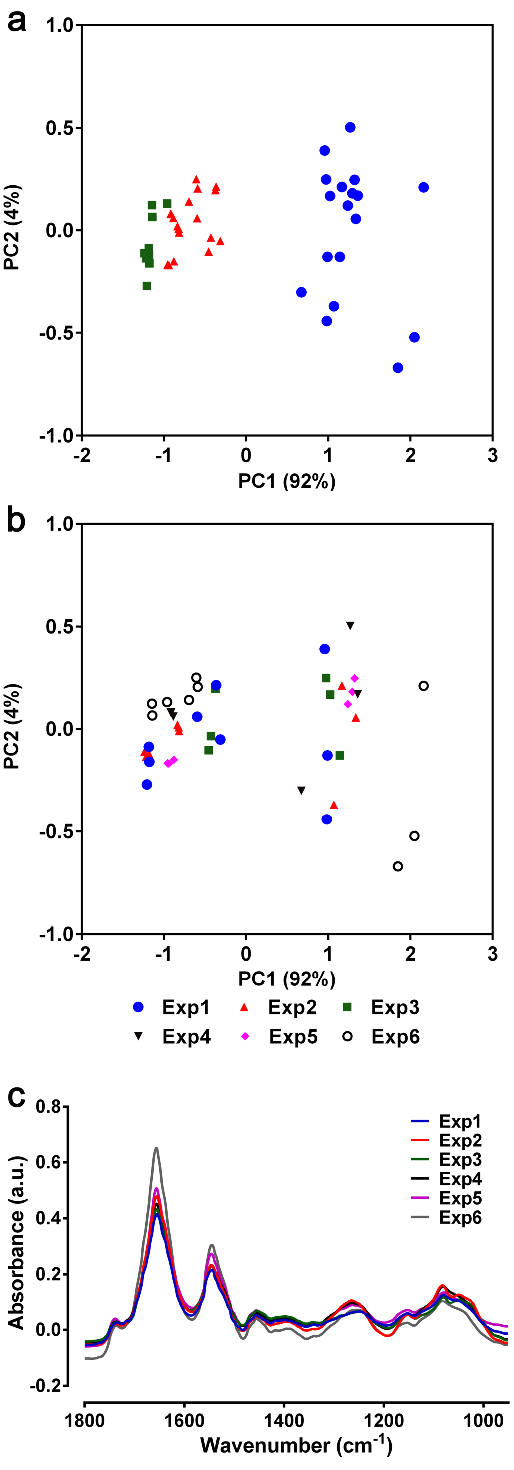

Fig. S2. Variability of FT-IR results between different experiments. Identical PCA score plots of EMSC2 normalised cut-down FTIR spectra generated from *C. reinhardtii* grown in either standard TAP, Low P TAP, or Low N TAP media, either with different growth media highlighted (a) or with different experiments highlighted (b). Mean spectra from 3 replicates generated from *C. reinhardtii* grown in TAP medium for each of the six independent experiments (c).

Supplementary Figure 3

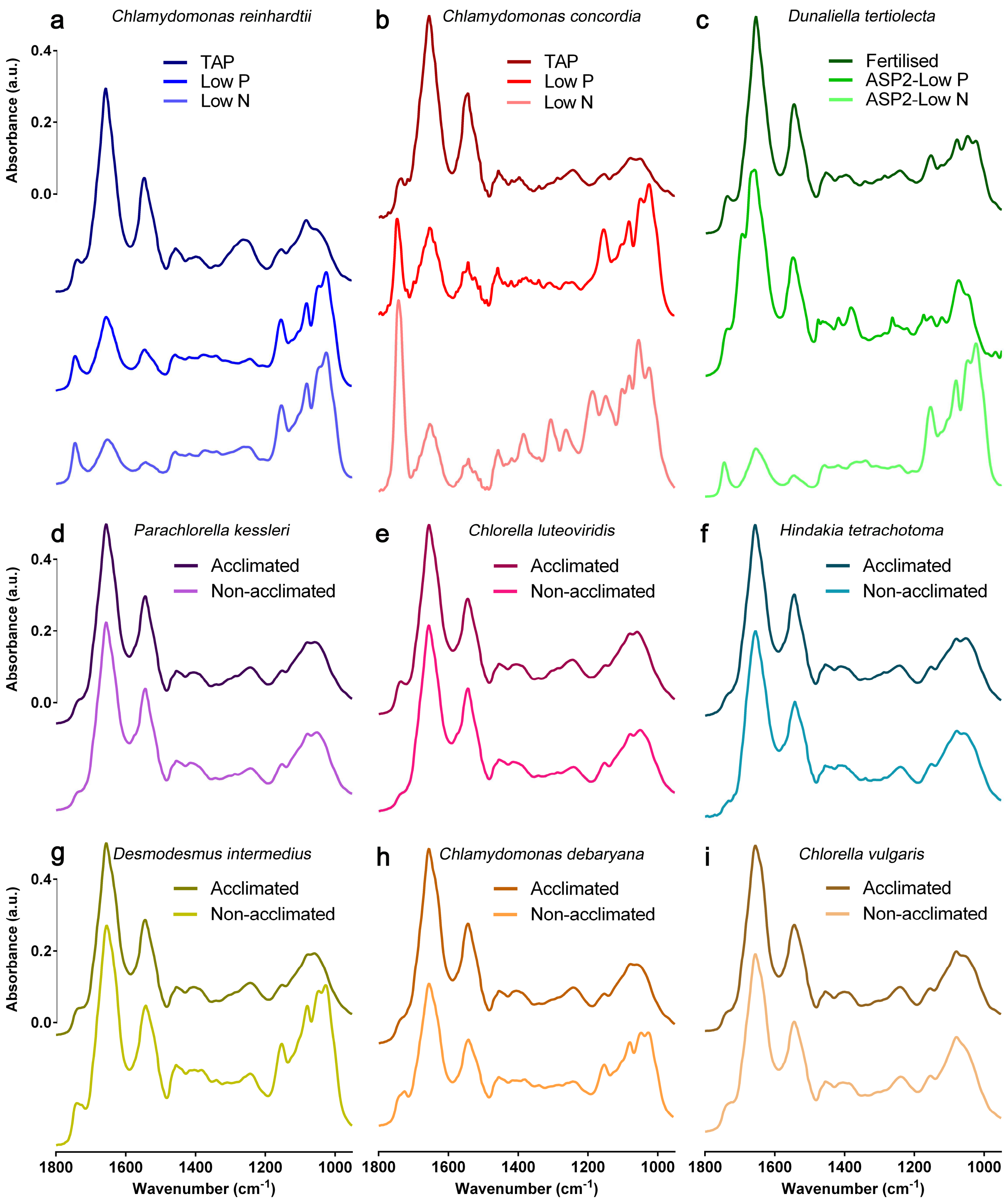

Fig. S3. Comparison of mean cut-down FT-IR spectra, following EMSC2 normalisation, generated from nine algal species grown for 7 days in non-stressed and stressed growth conditions, including *C. reinhardtii* and *C. concordia* grown in TAP, Low-P TAP, Low-N TAP media, *D. tertiolecta* grown in fertilised seawater, Low-P ASP2 and Low-N ASP2 media, and six other species that had been acclimated or non-acclimated for growth in wastewater. Spectra are averaged from 3-18 replicate spectra for each species and growth treatment.

Supplementary Figure 4

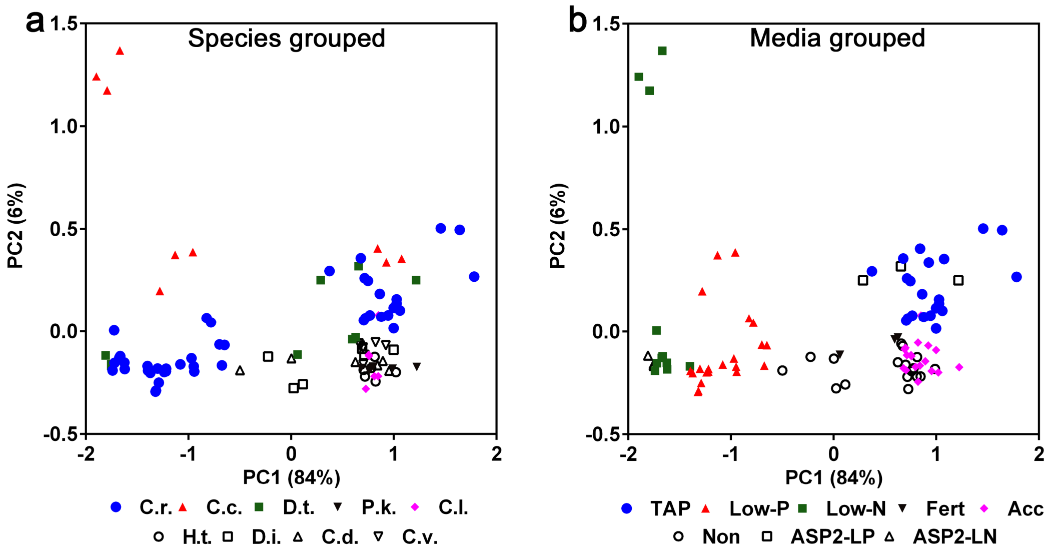

Fig. S4. Identical PCA scores plots of EMSC2 normalised cut-down FT-IR spectra of nine algal species grown for 7 days in non-stressed and stressed growth conditions. (a) Different species are highlighted using different symbols: C.r. = *C. reinhardtii,* C.c. = *C. concordia,* D.t. = *D. tertiolecta,* P.k. = *P. kessleri,* C.l. = *C. luteoviridis,* H.t. = *H. tetrachotoma,* D.i. = *D. intermedius,* C.d. = *C. debryana,* C.v. = *C. vulgaris.* (b) The same plot is shown but different media conditions are highlighted using different symbols: TAP, Low-P TAP, Low-N TAP, Fert = fertilised seawater, Acc = acclimated strains in wastewater, Non = non-acclimated strains in wastewater, ASP2-LP = Low-P ASP2, ASP2-LN = Low-N ASP2.

Supplementary Figure 5

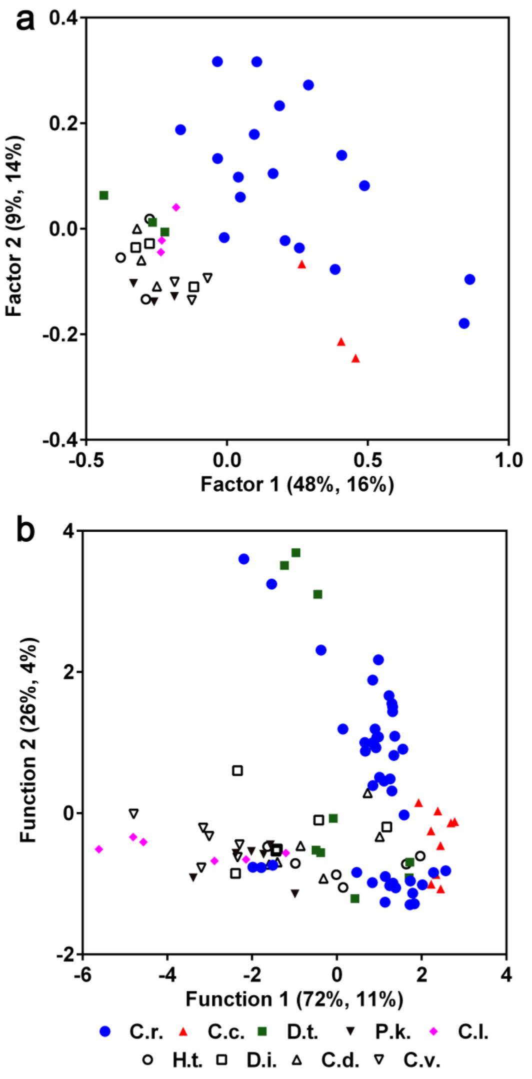

Fig. S5. PLS regression analysis scores plots of EMSC2 normalised cut-down FT-IR spectra of nine algal species grown for 7 days in non-stressed conditions (a) and a combined plot of the species grown in non-stressed and stressed growth conditions (b). Different species are highlighted using different symbols as described in Fig. S4.
